# Comparison between Second- and Third-Generation PTH Assays during Minimally Invasive Parathyroidectomy (MIP)

**DOI:** 10.1155/2020/5230985

**Published:** 2020-03-16

**Authors:** Marie-Hélène Gannagé-Yared, Nada Younès, Anne-Sophie Azzi, Ghassan Sleilaty

**Affiliations:** ^1^Endocrinology Department, Faculty of Medicine, Saint Joseph University, Beirut, Lebanon; ^2^Department of Biostatistics, Faculty of Medicine, Saint-Joseph University, Beirut, Lebanon; ^3^Clinical Research Center, Faculty of Medicine, Saint-Joseph University, Beirut, Lebanon

## Abstract

**Methods:**

112 patients (of which 72.3% females) underwent MIP by the same surgeon. Age, sex, body mass index (BMI), pre- and postoperative serum calcium, creatinine, 25(OH)D levels, PTH at baseline (PTH T0), and PTH at 10 minutes after adenoma resection (PTH T10) were recorded. Both PTH 2G and PTH 3G assays were assessed using the Diasorin assays.

**Results:**

The mean age was 56.1 ± 14.7 years. Mean value of BMI, preoperative calcium, 25(OH)D, and CKD-EPI-eGFR were, respectively, 26.8 ± 4.8 kg/m^2^, 110.9 ± 7.9 mg/L, 19.3 ± 9.2 ng/mL, and 88.6 ± 25.6 mL/min/1.73 m^2^. PTH 2G and PTH 3G assays were well correlated at PTH T0 and PTH T10 (respectively, correlation coefficient 0.74 and 0.72 for intraclass correlation type 3). The median PTH fall was, respectively, of 79.9% and 82.5% for PTH 2G and PTH 3G. Multivariate analysis using the combined PTH 2G and PTH 3G as a dependent variable with 2 repeated measurements (at PTH 0 and PTH 10) showed a significant effect of preoperative calcium on IOPTH fall (*p*=0.001, effect size 0.13), while no significant effects were observed for sex, age, BMI, and 25(OH)D.

**Conclusion:**

PTH 2G and PTH 3G assays resulted in a similar drop in IOPTH values. Elevated preoperative calcium levels are the only independent predictor of IOPTH decline. Further studies are needed to determine other factors that can influence PTH kinetics.

## 1. Introduction

Primary hyperparathyroidism (PHPT) is most frequently caused by a single adenoma localized in one of the parathyroid glands [1]. Surgery remains the only definitive cure of the disease. Previously managed by bilateral surgical neck exploration, PHPT is nowadays treated by minimally invasive parathyroidectomy (MIP). This change was primarily driven by the accuracy of preoperative localization tests allowing a unilateral neck exploration with a limited operative time, sometimes using just a cervical block with sedation [1–3]. The incorporation of intraoperative measurements of PTH (IOPTH) has facilitated MIP and reduced the need for further unnecessary explorations [4]. The Miami criterion for a successful parathyroidectomy was defined as a fall of IOPTH by more than 50% of its initial value, within 10 to 15 minutes after removal of the adenoma [5]. The IOPTH value drawn at 10 minutes following parathyroidectomy is the most accurate predictor of a successful MIP [6, 7]. Age, impaired renal function [8], race (African American vs. others) [9], and high BMI [10] were shown to be negative predictors of IOPTH decline following MIP, while low 25 hydroxyvitamin D (25(OH)D) levels have been shown to either increase IOPTH drop [11] or have no effect [12, 13].

Different generations of parathormone (PTH) assays are present in the market. The older ones, called “intact” PTH or second-generation PTH assays (PTH 2G) cross-react with an N-terminal truncated PTH fragment called (7–84) PTH or non-(1–84) PTH [14–17]. The more recent ones called third-generation assays (PTH 3G) do not detect the non-(1–84) PTH but measure a post-translational form called amino-PTH [18, 19]. Despite lower values in PTH 3G assays compared to PTH 2G (approximately 30 to 50% lower) [17, 20–22], both assays demonstrated a strong correlation in patients with normal renal function [20, 23] and on hemodialysis [22, 24], even if the difference between both methods increases when PTH values are high [23, 24].

It has been shown that during MIP a greater IOPTH drop was observed 10 minutes after excision in the PTH 3G compared to the PTH 2G assay [25, 26] in both primary [26] and secondary hyperparathyroidism [25]. Because of the lack of consistent data on this subject, we aimed to compare both PTH assays during MIP in patients with an estimated Chronic Kidney Disease Epidemiology Collaboration (CKD-EPI) equation > 30 mL/min/1.73 m^2^ and to identify the predictors of IOPTH decline.

## 2. Materials and Methods

### 2.1. Subjects

One hundred and twelve patients underwent MIP. The MIP surgeries were performed by the same surgeon at the department of general surgery of Hotel-Dieu de France university hospital, Beirut. MIP was performed only in cases with clearly positive localization on ultrasound or scan. Subjects with a family history of hyperparathyroidism or a personal or family history of multiple endocrine neoplasia or recurrent hyperparathyroidism after surgery were also excluded.

The following clinical data were retrospectively collected from the patients' records: age, sex, weight, height, body mass index (BMI) (calculated as weight in kilograms (kg) over the square of height in square meter (m^2^)), history of diabetes or hypertension, and bisphosphonate use. Prior to surgery, PHPT was diagnosed according to the following criteria: combined elevation of PTH and calcium levels for the hypercalcemic PHPT; elevated PTH level combined with a normal calcium and 25(OH)D levels (25(OH)D ≥ 20 ng/mL) for normocalcemic PHPT. Normocalcemic PHPT was found in 9 subjects (8% of the sample).

### 2.2. Surgical Procedure

Surgery was performed under local anesthesia (0.5–1% xylocaine with 1 : 200,000 adrenaline) assisted with an intravenous sedation. Propofol was given intravenously for sedation with titration according to the need of the patient. In addition, an intravenous bolus of fentanyl of 50 to 100 *μ*g was given before the incision to insure analgesia. A focused lateral approach through a 2 cm transverse skin mini incision was done directly over the parathyroid gland localized by preoperative imaging. Then the sternomastoid muscle was mobilized laterally, and the identified adenoma was removed and sent to pathology for extemporaneous analysis. The surgery time was 30 ± 8 min (range 15–55 min).

### 2.3. Laboratory Analysis

#### 2.3.1. Biochemistry Assessment

Fasting serum preoperative calcium, phosphorus, and creatinine levels were repeated on the day of the surgery between 08 : 00 and 09 : 00 AM and measured on dry chemistry by using a Kodak automate. Postoperatively on day 1, fasting calcium and phosphorus were also measured. The respective normal values for calcium and phosphorus were 2.1–2.56 mmol/L and 0.84–1.45 mmol/L. In addition to creatinine measurements, CKD-EPI equation was used for glomerular filtration rate estimation (eGFR). Results are expressed in mL/min/1.73 m^2^.

#### 2.3.2. PTH and 25(OH)D Measurements

PTH was measured intraoperatively at baseline (preincision or PTH T0) and at 10 minutes following surgical removal of the parathyroid adenoma (PTH T10), with both PTH 2G and PTH 3G assays. The PTH 2G was measured using the chemiluminescent Diasorin assay on the Liaison automate (Stillwater, USA). This method uses a monoclonal antibody specific for the central and C-terminal part of the molecule (amino acids 34–84) and a second polyclonal antibody recognizing the N-terminal part of the PTH. It recognizes both the PTH 1–84 and the peptide 7–84. The lower limit of detection is 3 pg/mL and the measurement range between 3 and 1900 pg/mL. The PTH 3G was also measured with a chemiluminescent Diasorin assay on the Liaison automate (Stillwater, USA). This assay allows the determination of the PTH 1–84 without cross-reactivity with the PTH 7–84 fragment, using two polyclonal antibodies, the first one specific to the N-terminal extremity of the peptide and the second specific to the C-terminal part ensuring 100% specificity for the whole PTH molecule. The minimal detectable level is 1.7 pg/mL. The respective normal values for healthy subjects are 7 to 36 pg/mL for PTH 3G assay and 11 to 62 pg/mL for PTH 2G. 25-(OH)D was measured using the Diasorin chemiluminescent automate Liaison (Stillwater, USA). Measurements of PTH 2G and 25(OH)D were delayed and measured on serum previously frozen at −80°C.

#### 2.3.3. Statistical Analysis

The distributions of quantitative variables were assessed with Q-Q plots. Shapiro–Wilk and Kolmogorov–Smirnov tests were used to check departure from normality. Continuous variables overtly nonnormal were expressed as median with its interquartile range (1^st^ quartile–3^rd^ quartile). Continuous variables not departing from normality were expressed as mean and its standard deviation. PTH assay distributions are expectedly heavily skewed motivating an inverse normal transform (INT) approach, using Van der Waerden ranks. A 2-way MANCOVA model was applied, using the INT of PTH 2G and PTH 3G assays as a combined dependent variable for multivariate analysis, followed by INT of PTH 2G and PTH 3G separately for post-MANCOVA univariate analysis. Two repeated measurements were specified (at baseline PTH T0 and at 10 min PTH T10), gender was taken as between-subject independent factor, and age, CKD-EPI-eGFR, BMI, preoperative calcium, and 25(OH)D levels were taken as covariates. Type III sum of squares was used, and a 2-way time by factor interactions were included in the model.

Agreement between the PTH 2G and PTH 3G assays was calculated using intraclass correlation coefficient type 3 (ICC [3], 95% confidence provided), assuming a 2-way mixed effects model where the subjects' effects are random, and measures' effects are fixed. Results were considered statistically significant for *p* values under 0.05.

Analyses were run using SPSS software (IBM Corp. Released 2013, SPSS Statistics for Windows Version 22.0, Armonk, NY).

## 3. Results

A total of 112 patients were included in this study, with a mean age of 56.1 ± 14.7 years (range 23–86 years). 72.3% of the subjects were females, 17.9% had diabetes, 42.9% had hypertension, and 6.3% were taking bisphosphonates. The mean BMI was 26.8 ± 4.8 kg/m^2^ (range 18.6–44.9 kg/m^2^).

### 3.1. Biochemical Measurements

The respective pre- and postoperative mean calcium levels were 110.9 ± 7.9 (range 97.2–156 mg/L) and 90.7 ± 7.4 mg/L (range 70.8–114 mg/L). The mean CKD-EPI-eGFR was 88.6 ± 25.6 mL/min/1.73 m^2^ (range 30.0–164.2 mL/min/1.73 m^2^) with 95 patients (84.8%) having a CKD-EPI-eGFR ≥ 60 mL/min/1.73 m^2^ and 17 (15.2%) between 30 and 60 mL/min/1.73 m^2^. The mean 25(OH)D level was 19.3 ± 9.2 ng/mL (range 4–44.6 ng/mL).

### 3.2. IOPTH Drop in PTH 2G and PTH 3G Assays

The median and interquartile values of baseline PTH (PTH T0) using the PTH 2G and PTH 3G assays were, respectively, 121.5 pg/mL (89.5–172.5 pg/mL) (range 17.4–992 pg/mL) and 64.2 pg/mL (48.7–90.5 pg/mL) (range 27.8–538 pg/mL). There was no difference in PTH 2G and PTH 3G between subjects who took bisphosphonates and those who did not (*p*=0.990 and *p*=0.696, respectively). Ten minutes after the adenoma removal, the median and interquartile values of PTH (PTH T10) were 24.1 pg/mL (16.8–37.91 pg/mL) (range 3.3–172 pg/mL) for PTH 2G and 12.3 pg/mL (8.4–19.2 pg/mL) (range 4–119 pg/mL) for PTH 3G. The median IOPTH drop was 79.9% (71.4%–85.8%) for the PTH 3G and 82.5% (72.9%–87.9%) for the PTH 2G (*p*=0.566) (data shown in Table 1).

There was a good agreement between the 2 assays for both baseline PTH T0 and PTH T10 (ICC [3] 0.735 (95% CI 0.311–0.871) and 0.722 (95% CI 0.157–0.876), respectively).

### 3.3. Uni- and Multivariate Analysis Studying the Relationship between Baseline PTH Values and Other Variables

MANCOVA analysis revealed that 25(OH)D and preoperative calcium levels are independently associated with the combined PTH 2G and PTH 3G assay variable (*p*=0.016, effect size 0.094 for 25(OH)D; *p*=0.017, effect size 0.093 for preoperative calcium levels), while the other variables did not achieve significance (age, BMI, and CKD-EPI-eGFR) (data not shown in tables).

In univariate analysis following MANCOVA, studying separately PTH 2G and PTH 3G, the effect of 25(OH)D levels was similar for PTH 2G and 3G (for PTH 2G, *p*=0.013, effect size 0.070; for PTH 2G, *p*=0.004, effect size 0.094). The preoperative calcium was also significant for the PTH 2G (*p*=0.009, effect size 0.078) and for PTH 3G (*p*=0.004, effect size 0.092) (Table 2).

### 3.4. Uni- and Multivariate Analysis Studying the Predictors of IOPTH Fall

MANCOVA of the combined PTH 2G and PTH 3G as a dependent variable with 2 repeated measurements (at PTH T0 and PTH T10) showed a significant effect of preoperative calcium on IOPTH fall (*p*=0.007, effect size 0.110), with no significant effects of the other factors (age, sex, CKD-EPI-eGFR, BMI, and 25(OH)D) (Table 3).

The univariate analysis studying separately PTH 2G and PTH 3G, showed that only the preoperative calcium levels had a significant effect on IOPTH fall in both assays (*p*=0.015, effect size 0.067 for PTH 2G; *p*=0.002, effect size 0.109 for PTH 3G) (Table 3).

**Figure 1:**
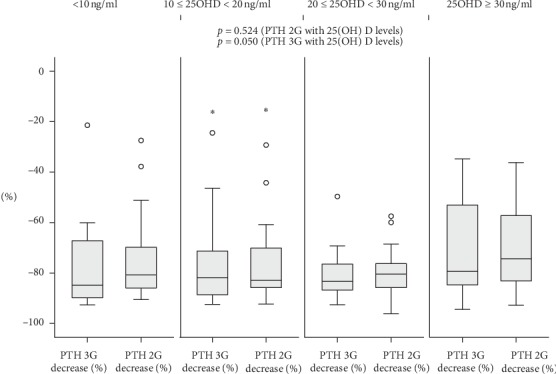
Effect of 25(OH)D on IOPTH decline, using PTH 2G and PTH 3G assays. PTH 2G, 2^nd^ generation PTH assay; PTH 3G, 3^rd^ generation PTH assay; IOPTH, Intraoperative PTH decline. Drop of PTH 2G and PTH 3G results are shown as boxplots. *p* values correspond to those of the Spearman correlation coefficient between PTH 2G and PTH 3G on one hand and 25(OH) D on the other hand. Small circles correspond to outliers, and stars correspond to extreme outliers.

**Figure 2:**
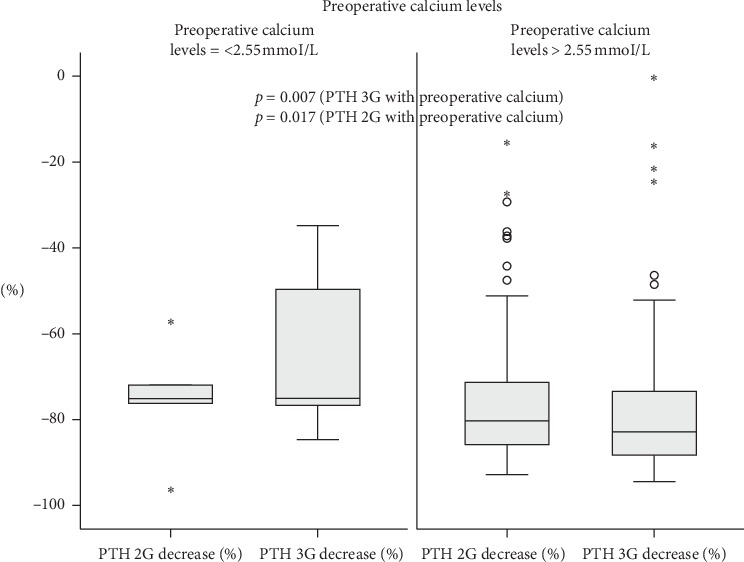
Effect of preoperative calcium (preoperative calcium ≤ 2.55 mmol/L vs >2.55 mmol/L) on IOPTH decline, using PTH 2G and PTH 3G assays. PTH 2G, 2^nd^ generation PTH assay; PTH 3G, 3^rd^ generation PTH assay; IOPTH, Intraoperative PTH decline. Drop of PTH 2G and PTH 3G results are shown as boxplots. *p* values correspond to those of the Spearman correlation coefficient between PTH 2G and PTH 3G on one hand and preoperative calcium levels on the other hand. Small circles correspond to outliers and stars correspond to extreme outliers.

When analyzed separately according to 25(OH)D levels, the magnitude of the PTH drop was inversely correlated with 25(OH)D levels for PTH 3G (Spearman's rho = −0.076, *p*=0.050) but not for PTH 2G (Spearman's rho = −0.025, *p*=0.524), as shown in Figure 1. The magnitude of PTH drop was also correlated with the preoperative calcium level for both PTH 2G and PTH 3G (Spearman's rho = 0.254, *p*=0.007 for PTH 3G; Spearman's rho = 0.228, *p*=0.017 for PTH 2G) as shown in Figure 2.

## 4. Discussion

The purpose of our study was to compare the IOPTH decline using 2 different PTH assays in 112 patients undergoing MIP and to determine the predictors of this decline. Our results showed a respective IOPTH fall of 79.9% and 82.5% for the PTH 3G and PTH 2G assay, with no significant difference between both assays. Two previous groups, the first one from Japan [25, 26] and the second one from Austria [27, 28], reported a quicker drop of the IOPTH using a PTH 3G assay compared to a PTH 2G one. The difference between both assays could be explained by the cross-reactivity of PTH 2G assays with non-(1–84) PTH fragments of longer half-lives [26], resulting in a slower IOPTH drop. However, the abovementioned studies were mainly performed on a small sample of subjects with renal hyperparathyroidism (rHPT) [25, 27, 28], either secondary to hemodialysis [25, 27, 28] or following renal transplantation [27, 28]. The only study that included a subgroup of subjects with PHPT was the Japanese one [26] in which 74 patients with PHPT were enrolled. The authors of the latter study found a greater difference in IOPTH fall between both assays in hemodialysis subjects compared to subjects with PHPT, suggesting that renal function affects the IOPTH drop. The difference between these studies and our study could be explained by the normal renal function in the majority of the current sample (84.8% of our sample had a CKD-EPI-eGFR ≥ 60 mL/min/1.73 m^2^), the use of different PTH assays in our study, and more importantly, the timing of sampling during the surgery. In fact, in both the Japanese [25, 26] and Austrian studies [27, 28], the Quick Roche Elecsys PTH assay was used for the 2G assay, while either the Scantibodies Laboratory assay [25, 26] or the Nichols assay [27, 28] was used for the 3G assay. Subsequently, one could speculate that different PTH kinetics exist between these 3 assays and the Diasorin assays that were used in our study. In fact, it is possible that the short turnout of the Quick Roche Elecsys PTH 2G assay, which is of 6 minutes, does not allow the achievement of the same IOPTH fall observed with the PTH 3G assay. In addition, the IOPTH value at 10 minutes after excision of adenoma was used in our study as a predictive value for surgical cure, as established by the Miami criterion [5]. Collecting a sample at a later timing, for example at 15 minutes [25, 26] or even later at a maximum of 50 minutes [26] could have led to a divergence between both assays.

We then studied the effect of gender, age, BMI, CKD-EPI-eGFR, preoperative calcium, and 25(OH)D levels on IOPTH decline. On one hand, we found that the only predictor of IOPTH fall was the preoperative calcium level, the presence of elevated levels being associated with a greater IOPTH decrease, no matter the used assay. Those results are in line with two other studies demonstrating that subjects with the normocalcemic variant of PHPT have a slower drop in their IOPTH [10, 29]. On the other hand, gender, age, and BMI did not affect the IOPTH decline. Similarly to our results, another study did not find that gender influences the IOPTH drop [10]. In addition, in our previous report [8], age but not gender was a predictor of IOPTH decline, since an inverse relationship between age and IOPTH was noted. Also, another study showed an inverse relation between BMI and IOPTH in patients younger than 55 years [10]. Of note, we found that the CKD-EPI-eGFR was not a predictor of the IOPTH contrary to our previous study demonstrating an inverse relationship between the CKD-EPI-eGFR and IOPTH decline [8]. The reason behind these different results could be related to other characteristics of our population (age being slightly younger age and majority of patients having a normal renal function) or to different used PTH assays.

Finally, we analyzed the effect of 25(OH)D levels on the IOPTH decline. In addition to being inversely correlated with PTH values in uni- and multivariate analysis, 25(OH)D was found to have an effect on IOPTH decline. Because vitamin D deficiency stimulates parathyroid hyperplasia and is associated with larger parathyroid adenomas and higher levels of PTH before and after surgery for PHPT [30], one could speculate that the IOPTH decline is slower in vitamin D deficient patients [31, 32]. However, results of studies looking at the impact of 25(OH)D status on IOPTH kinetics are controversial. While one study has shown an inverse relationship between 25(OH)D levels and IOPTH decline [11], others did not find the same results [12, 13, 33–35]. We also found a significant drop in IOPTH regardless of 25(OH)D classes. The drop in PTH 3G was significantly and inversely correlated with 25(OH)D, a finding that is in line with the Agarwal et al. study [11], but the small effect size precludes any meaningful inference, noting that in univariate F tests following MANCOVA (Table 3), the effect of 25(OH)D was not found to be significant.

The strength of our study is that it is the first one to compare PTH 2G and PTH 3G assays in a large sample of subjects undergoing a MIP. Unfortunately, both the Diasorin PTH 2G and PTH 3G used assays in our study need an approximate complete turnout of 50 minutes compared to the only two other available QUICK PTH assays (Siemens Immulite® Turbo PTH and Roche Elecsys) which need less than 15 minutes. Subsequently, the surgical decision-making process requires a longer time during which the patient is still in the operative room. This is the reason why an IOPTH sampling was not performed at a later time than 10 minutes in our institution. Our surgeon relies solely on the extemporaneous pathology in order to extend the surgical procedure. Nevertheless, our results confirm the fact that developing a quick third-generation assay may not be an added value compared to the second-generation assays.

## 5. Conclusion

In conclusion, our study shows that PTH 2G and PTH 3G assays resulted in a similar drop in IOPTH values. Eelevated preoperative calcium level was an independent predictor of IOPTH while age, sex, BMI, CKD-EPI-eGFR, and 25 (OH)D levels were not. Our results suggest that current criteria for IOPTH monitoring are applicable regardless of the used PTH assay and the clinical characteristics of the patients.

## Figures and Tables

**Table 1:** Pre- and postoperative values of the assessed biochemical variables.

Preoperative calcium (mg/L)	110.9 ± 7.9
Postoperative calcium (mg/L)	90.7 ± 7.4
Preoperative phosphorus (mmol/L)	1.00 ± 0.16
Postoperative phosphorus (mmol/L)	1.26 ± 0.25
CKD-eGFR (ml/min/1.73 m^2^)	88.6 ± 25.6
PTH 3G T0 (pg/mL)	64.2 [48.7–90.5]
PTH 3G T10 (pg/mL)	12.3 [8.4–19.2]
PTH 2G T0 (pg/mL)	121.5 [89.5–172.5]
PTH 2G T10 (pg/mL)	24.1 [16.8–37.91]
Percentage of IOPTH 2G decrease	79.9% [71.4%–85.5%]
Percentage of IOPTH 3G decrease	82.5% [72.9%–87.9%]
25(OH)D level (ng/mL)	19.3 ± 9.2

Data are expressed as mean ± SD or median and its interquartile range.

**Table 2:** Univariate analysis studying the relationship between baseline PTH values and other variables (a) computed at alpha 5%).

Univariate between subjects F tests	Variable	*p* value	Effect size (partial eta squared)	Power
Intercept	PTH_3G	0.035	0.052	0.565
	PTH_2G	0.058	0.042	0.476

Age	PTH_3G	0.109	0.030	0.360
	PTH_2G	0.370	0.009	0.145

CKD-EPI-eGFR	PTH_3G	0.974	0.000	0.050
	PTH_2G	0.583	0.004	0.085

BMI	PTH_3G	0.690	0.002	0.068
	PTH_2G	0.225	0.017	0.227

25(OH)D	PTH_3G	0.004	0.094	0.836
	PTH_2G	0.013	0.070	0.709

Preoperative calcium	PTH_3G	0.004	0.092	0.825
	PTH_2G	0.009	0.078	0.758

Gender	PTH_3G	0.979	0.000	0.050
	PTH_2G	0.788	0.001	0.058

**Table 3:** Uni- and multivariate analysis studying the predictors of IOPTH fall (a) computed at alpha 5%).

	Wilk's lambda	*p* value	Effect size (partial eta squared)	Power

Multivariate tests within subjects				
Time	0.908	0.018	0.092	0.727
Time *∗* age	0.991	0.683	0.009	0.110
Time *∗* CKD-EPI-eGFR	0.980	0.427	0.020	0.193
Time *∗* BMI	0.981	0.443	0.019	0.187
Time *∗* 25(OH)D	0.987	0.583	0.013	0.137
Time *∗* preoperative calcium	0.890	0.007	0.110	0.816
Time *∗* gender	0.949	0.110	0.051	0.449

Univariate within subjects F tests				
	Variable			

Time	PTH_3G	0.005	0.091	0.822
	PTH_2G	0.013	0.070	0.708
Time *∗* age	PTH_3G	0.477	0.006	0.109
	PTH_2G	0.381	0.009	0.140
Time *∗* CKD-EPI-eGFR	PTH_3G	0.393	0.009	0.136
	PTH_2G	0.206	0.019	0.242
Time *∗* BMI	PTH_3G	0.203	0.019	0.245
	PTH_2G	0.331	0.011	0.162
Time *∗* 25(OH)D	PTH_3G	0.944	0.000	0.051
	PTH_2G	0.596	0.003	0.082
Time *∗* preoperative calcium	PTH_3G	0.002	0.109	0.890
	PTH_2G	0.015	0.067	0.687

## Data Availability

The excel data used to support the findings of this study are available from the corresponding author upon request.

## Abbreviations

PTH: Parathormone
25(OH)D: 25-hydroxyvitamin D
IOPTH: Intraoperative parathormone.

## References

[1] Insogna K. L. (2018). Primary hyperparathyroidism. *New England Journal of Medicine*.

[2] Sackett W. R., Barraclough B., Reeve T. S., Delbridge L. W. (2002). Worldwide trends in the surgical treatment of primary hyperparathyroidism in the era of minimally invasive parathyroidectomy. *Archives of Surgery*.

[3] Udelsman R. (2002). Six hundred fifty-six consecutive explorations for primary hyperparathyroidism. *Annals of Surgery*.

[4] Richards M. L., Thompson G. B., Farley D. R., Grant C. S. (2011). An optimal algorithm for intraoperative parathyroid hormone monitoring. *Archives of Surgery*.

[5] Irvin G. L., Solorzano C. C., Carneiro D. M. (2004). Quick intraoperative parathyroid hormone assay: surgical adjunct to allow limited parathyroidectomy, improve success rate, and predict outcome. *World Journal of Surgery*.

[6] Irvin G. L., Dembrow V. D., Prudhomme D. L. (1993). Clinical usefulness of an intraoperative “quick parathyroid hormone” assay. *Surgery*.

[7] Sokoll L. J., Drew H., Udelsman R. (2000). Intraoperative parathyroid hormone analysis: a study of 200 consecutive cases. *Clinical Chemistry*.

[8] Gannagé-Yared M. H., Abboud B., Amm-Azar M. (2009). Predictors of intra-operative parathyroid hormone decline in subjects operated for primary hyperparathyroidism by minimally invasive parathyroidectomy. *Journal of Endocrinological Investigation*.

[9] Cisco R. M., Kuo J. H., Ogawa L. (2012). Impact of race on intraoperative parathyroid hormone kinetics. *Archives of Surgery*.

[10] Leiker A. J., Yen T. W. F., Eastwood D. C. (2013). Factors that influence parathyroid hormone half-life. *JAMA Surgery*.

[11] Agarwal G., Sadacharan D., Ramakant P., Shukla M., Mishra S. K. (2012). The impact of vitamin D status and tumor size on the intraoperative parathyroid hormone dynamics in patients with symptomatic primary hyperparathyroidism. *Surgery Today*.

[12] Statham M. M., Watts N. B., Steward D. L. (2007). Intraoperative PTH: effect of sample timing and vitamin D status. *Otolaryngology—Head and Neck Surgery: Official Journal of American Academy of Otolaryngology-Head and Neck Surgery*.

[13] Graves C. E., McManus C. M., Chabot J. A., Lee J. A., Kuo J. H. (2019). Vitamin D does not affect intraoperative parathyroid hormone kinetics: a mixed linear model analysis. *Journal of Surgical Research*.

[14] D’Amour P., Brossard J.-H., Rousseau L. (2005). Structure of non-(1-84) PTH fragments secreted by parathyroid glands in primary and secondary hyperparathyroidism. *Kidney International*.

[15] Lepage R., Roy L., Brossard J.-H. (1998). A non-(1-84) circulating parathyroid hormone (PTH) fragment interferes significantly with intact PTH commercial assay measurements in uremic samples. *Clinical Chemistry*.

[16] Michelangeli V. P., Heyma P., Colman P. G., Ebeling P. R. (1997). Evaluation of a new, rapid and automated immunochemiluminometric assay for the measurement of serum intact parathyroid hormone. *Annals of Clinical Biochemistry: An International Journal of Biochemistry and Laboratory Medicine*.

[17] Smit M. A., van Kinschot C. M. J., van der Linden J., van Noord C., Kos S. (2019). Clinical guidelines and PTH measurement: does assay generation matter?. *Endocrine Reviews*.

[18] Souberbielle J. C., Boudou P., Cormier C. (2008). Lessons from second- and third-generation parathyroid hormone assays in primary hyperparathyroidism. *Journal of Endocrinological Investigation*.

[19] D’Amour P., Brossard J.-H., Rousseau L., Roy L., Gao P., Cantor T. (2003). Amino-terminal form of parathyroid hormone (PTH) with immunologic similarities to hPTH(1-84) is overproduced in primary and secondary hyperparathyroidism. *Clinical Chemistry*.

[20] Boudou P., Ibrahim F., Cormier C., Chabas A., Sarfati E., Souberbielle J.-C. (2005). Third- or second-generation parathyroid hormone assays: a remaining debate in the diagnosis of primary hyperparathyroidism. *The Journal of Clinical Endocrinology & Metabolism*.

[21] Ljungdahl N., Haarhaus M., Linder C., Magnusson P. (2006). Comparison of 3 third-generation assays for bio-intact parathyroid hormone. *Clinical Chemistry*.

[22] Melamed M. L., Eustace J. A., Plantinga L. C. (2008). Third-generation parathyroid hormone assays and all-cause mortality in incident dialysis patients: the CHOICE study. *Nephrology Dialysis Transplantation*.

[23] Dupuy A. M., Bargnoux A. S., Morena M. (2018). Moving from the second to the third generation Roche PTH assays: what are the consequences for clinical practice?. *Clinical Chemistry and Laboratory Medicine (CCLM)*.

[24] Gannagé-Yared M.-H., Farès C., Ibrahim T., Rahal Z. A., Elias M., Chelala D. (2013). Comparison between a second and a third generation parathyroid hormone assay in hemodialysis patients. *Metabolism*.

[25] Yamashita H., Cantor T., Uchino S. (2005). Sequential changes in plasma intact and whole parathyroid hormone levels during parathyroidectomy for secondary hyperparathyroidism. *World Journal of Surgery*.

[26] Yamashita H., Gao P., Cantor T. (2004). Comparison of parathyroid hormone levels from the intact and whole parathyroid hormone assays after parathyroidectomy for primary and secondary hyperparathyroidism. *Surgery*.

[27] Kaczirek K., Prager G., Riss P. (2006). Novel parathyroid hormone (1-84) assay as basis for parathyroid hormone monitoring in renal hyperparathyroidism. *Archives of Surgery*.

[28] Bieglmayer C., Kaczirek K., Prager G., Niederle B. (2006). Parathyroid hormone monitoring during total parathyroidectomy for renal hyperparathyroidism: pilot study of the impact of renal function and assay specificity. *Clinical Chemistry*.

[29] Graves C. E., McManus C. M., Chabot J. A., Lee J. A., Kuo J. H. (2020). Biochemical profile affects IOPTH kinetics and cure rate in primary hyperparathyroidism. *World Journal of Surgery*.

[30] Carsote M., Paduraru D., Nica A., Valea A. (2016). Parathyroidectomy: is vitamin D a player for a good outcome?. *Journal of Medicine and Life*.

[31] Carty S. E., Roberts M. M., Virji M. A., Haywood L., Yim J. H. (2002). Elevated serum parathormone level after “concise parathyroidectomy” for primary sporadic hyperparathyroidism. *Surgery*.

[32] Özbey N., Erbil Y., Ademoğlu E., Özarmağan S., Barbaros U., Bozbora A. (2006). Correlations between vitamin D status and biochemical/clinical and pathological parameters in primary hyperparathyroidism. *World Journal of Surgery*.

[33] Adler J. T., Sippel R. S., Chen H. (2010). 25-hydroxyvitamin D status does not affect intraoperative parathyroid hormone dynamics in patients with primary hyperparathyroidism. *Annals of Surgical Oncology*.

[34] Untch B. R., Barfield M. E., Dar M., Dixit D., Leight G. S., Olson J. A. (2007). Impact of 25-hydroxyvitamin D deficiency on perioperative parathyroid hormone kinetics and results in patients with primary hyperparathyroidism. *Surgery*.

[35] Singh D. N., Gupta S. K., Chand G. (2013). Intra-operative parathyroid hormone kinetics and influencing factors with high baseline PTH: a prospective study. *Clinical Endocrinology*.

